# The Effects of Visual Behavior and Ego-Movement on Foveated Rendering Performance in Virtual Reality

**DOI:** 10.1007/s42979-025-03885-7

**Published:** 2025-04-15

**Authors:** David Petrescu, Paul A. Warren, Zahra Montazeri, Gabriel Strain, Steve Pettifer

**Affiliations:** 1 Computer Science, University of Manchester, Oxford Road, Manchester, M13 9PL UK; 2 Division of Psychology, Communication and Human Neuroscience, Virtual Reality Research (VR2) Facility, University of Manchester, Oxford Road, Manchester, M13 9PL UK

**Keywords:** Foveated Rendering, Variable Rate Shading, Psychophysics, Movement, Attention, Visual Behavior, Virtual Reality

## Abstract

Despite the recent developments in VR, maintaining photorealism is difficult due to the increased bandwidth capabilities and computational resources required. To make VR more affordable, techniques such as Foveated Rendering (FR) offer promising ways to optimise rendering without compromising the user experience. Near-eye displays with 6DOF tracking enable users to freely move through the environment. This was previously impossible with traditional displays. This work aims to disentangle the effect of type of ego-movement (Active versus Implied) and task type (Simple Fixations versus a task involving Fixations, Discrimination, and Counting) on a dynamic FR method developed using Variable Rate Shading (VRS) (a quarter of the native shading rate is used in the visual periphery). We also explore if the aforementioned effects are consistent under different visual behaviours (visual search versus tracking). Results show that participants actively moving and performing complex tasks (during visual search) are less sensitive to degradation than in other conditions, with only 31.7% of the FOV required to be rendered using full sampling. Additionally, we provide evidence for how instances of visual pursuit might influence these results; in this case, only 29.3% of the FOV rendered using full sampling is tolerated by participants.

## Introduction

The recent release of affordable VR and Mixed Reality Head Mounted Displays (HMDs) equipped with powerful chips that can generate realistic Virtual Environments (VEs) means that immersive technologies are gaining more consumer interest [1]. However, rendering photorealistic environments in real-time requires extensive computation. The rapid advances in HMD resolutions and refresh rates mean that rendering and bandwidth requirements become proportionally more expensive. Therefore, operations typical of graphical applications such as shading require significant Graphical Processing Unit (GPU) computations during VR usage. Techniques such as perceptually-driven rendering can be used in order to optimise VR content without compromising the visual experience of the user. One such technique is Foveated Rendering (FR). Whilst the Human Visual System (HVS) is particularly adept at resolving fine detail in the foveal region (i.e. the focal point), it becomes less proficient as eccentricity increases due to the uneven distribution of retinal photoreceptors [2, 3]. FR methods are able to exploit these perceptual limits without affecting the overall experience and present a promising solution for decreasing the higher computational loads typical of immersive VR environments [4–6].

As most VR devices are equipped with 6-Degrees Of Freedom (6DOF) tracking, users are now commonly moving through a virtual environment via active movement (i.e. under their own steam; kinematic locomotion) [7]. However, passively moving via implied movement (e.g. simulated movement in a vehicle or using the controllers to navigate) is also common to overcome the limitations of restricted space. Furthermore, users are often intentionally engaged in a task (e.g. finding enemies, searching for items), but also tracking targets [8]. Concerning locomotion, there is evidence that active movement differs from passive movement due to the complexity of the neural processes involved [9]. More specifically, the interplay between vestibular, proprioceptive, visual, optical flow, and efference copy systems in active movement are required for a complete and accurate representation of the environment [10]. With respect to task engagement, the deployment of *attention* is required (see Section “Attention”). Based on previous work, we know that:

- Retinal motion and/or motion from self-induced movement cause a decrease in visual sensitivity to fine detail [11, 12].
- The visual behaviour of observers is task-dependent, influencing visual acuity, eye fixations, and/or tracking [8, 12].
- Variable Rate Shading (VRS) artefacts in a fixed-foveated algorithm are less conspicuous during active movement compared to implied movement [13].
- Attention can be modulated by varying task difficulty [14]. This modulates visual sensitivity [15], and thus affects the extent to which foveated graphics can be used [16].

The issues highlighted above suggest that there is the potential for further improvment of FR algorithms by considering user movement, task engagement, and the visual behaviour used to complete dynamic tasks. In particular, there may be greater scope for lower-quality rendering (and thus lower required levels of GPU computation) for users who are both moving and engaged in a task and these results might be dependent on the visual behaviour employed to complete the tasks. In the present study, we investigate this potential and compare results across observations which involve the deployment of different visual behaviours. In Experiment 1 (E1), we achieve this by using readily available information about user movement and task type in observations in which the visual behaviour is not enforced - a direct approach that does not rely on models of vision. In Experiment 2 (E2), the observations involve tracking a guiding sphere towards the task objects in order to explore if the results in E1 are consistent during visual pursuit.

## Background

### Foveated Rendering (FR)

Foveated graphics are a family of perceptually-driven rendering methods that rely on the uneven distribution of receptors across the retina [4, 17]. Consequently, visual properties change at different eccentricities from the focal point. More specifically, sensitivity to fine detail decreases as a function of retinal eccentricity. FR exploits this property by optimising rendering quality as a function of the falloff in visual acuity. As such, vision models of how different perceptual properties are modulated across the retina as a function of spatiotemporal sensitivity can be derived and used to enhance optimisation methods [18, 19]. Foveated graphics can be *gaze-contingent* (eye-tracked—degradation follows gaze) or *fixed* to the centre of the display. For a state-of-the-art report refer to Wang et al. [20].

FR is especially attractive for VR HMDs. Near-eye displays with eye-tracking capabilities are becoming more readily available and generally benefit from FR methods. In their work, Guenter et al. [4] approximated the retinal falloff in acuity to a linear function in Minimum Angle of Resolution (MAR) units (acuity reciprocal). In their implementation of FR, an aggressive degradation MAR slope resulted in a sampling performance that could be increased by a factor of five compared to non-FR methods. Other implementations have shown promising results for optimising shading [21, 22], path tracing [23], or geometrical LOD strategies [24, 25]. Models of vision accounting for the effects of eccentricity on perception have been used to derive visual difference predictors [26, 27] or to enhance foveated graphics whilst accounting for contrast sensitivity [28, 29]. Other applications of foveated graphics involve improving the user experience rather than optimising content. Examples include increasing the perceived dynamic range of the image [30] or achieving more efficient power consumption [31, 32].

In this study, we introduce a novel FR paradigm by showing that readily available information about movement, type of retinal motion, and cognitive load can be used to optimise a dynamic FR method implemented using Variable Rate Shading (see Section “Variable Rate Shading (VRS)” for more detail).

### Variable Rate Shading (VRS)

VRS [33] is a technology introduced on the Nvidia Turing architecture NVIDIA [33]. It enables granular and variable control of shading across different parts of an image. Conceptually, VRS has many similarities to coarse pixel shading—a technique used previously for shading optimisation as part of an FR algorithm in Vaidyanathan et al. [21]. The viewport image rendered in each frame is divided into 16$\times$16px tiles which can be shaded in different configurations. VRS does not influence the rasterisation process; this limits any potential additional aliasing. The shading rate of the 16$\times$16px tile can either have a uniform configuration: {2$\times$2, 4$\times$4} or a non-uniform one: {1$\times$2, 2$\times$1, 2$\times$4, 4$\times$2}. Note that more than one configuration can be used for a rendered image at any time as tiles are controlled independently. To minimise calls to the fragment shader, the final pixel appearance is derived from only one value based on the block of pixels in each configuration. It is important to mention that coarse shading results in visual artefacts which are scene-dependent. Certain textures (e.g. checkered patterns or high spatial resolution textures) are more affected by VRS. In our experiment, we use VRS4$\times$4 for maximum optimisation benefits.

### Attention

Recently, considerable research effort in the perceptual graphics community has shifted from low-level visual processing (e.g. contrast sensitivity) to higher-order processes such as visual attention. Attention is often characterised as a ‘spotlight’ or ‘variable zoom lens’ [34] for the focusing of cognitive resources [35].

The term *overt attention* typically refers to the process of directly orienting one’s gaze to objects of interest, whereas *covert attention* refers to the mechanisms of directing attention to a part (or potentially multiple parts) of the environment without moving the eyes. Covert attention is thought to precede overt attention and guide subsequent eye movements and fixations. It is important to note that during the deployment of attention, peripheral sensitivity is decreased in order to concentrate resources on the point of focus [15]. Moreover, there is evidence that there is a physiological change in the photoreceptors’ receptive field profile when a viewer is actively attending to a stimulus [36]. In the present work, we study how visual sensitivity changes during the voluntary monitoring of information and with the participant performing certain tasks employing endogenous, overt attention.

With respect to visual sensitivity, there is evidence that attending overtly to a focal point improves performance and acuity in the foveal region [37]. However, the presence of a task that requires increased mental load tends to significantly reduce one’s visual acuity [38]. In support of this, the deployment of attention is also thought to reduce contrast sensitivity across the retina [14]. Recently, Krajancich et al. [16] created an attention-aware model of contrast sensitivity. By running extensive user studies, they provided strong evidence that the presence of a task which requires the deployment of increased levels of attention (induced by rapid serial presentation of letters) significantly lowers the ability to resolve fine detail in the periphery (i.e. the tolerated degradation varies as a function of the attention required to complete a task). In this work, we aim to explore if different levels of attentional load during VR use result in a lower sensitivity to degradation caused by an FR method implemented using VRS. Moreover, we explore this in a dynamic environment where users are performing translational movements more akin to typical VR usage. We introduce a new methodology (see Section “Task”), expanding on Krajancich et al. [16], where we replace the rapid presentation of letters with presentations of 3D objects (see Section “Stimuli”).

### Movement and Optimisation

Retinal motion represents one of the main factors affecting visual acuity. Motion on the retina can typically arise due to eye movements, world movement, or ego-movement. With respect to the first, it has been shown that during visual pursuit or saccades, sensitivity is modulated [11, 12]. Regarding world movement, Suchow and Alvarez [39, 40] explored a phenomenon termed ‘silencing’ which highlights that the presence of motion in the background impairs the ability to discern between qualitative properties of the foreground stimuli, such as luminance, colour, size, and shape. During self-movement, peripheral acuity is reduced in order to prevent retinal slips [41] and to preserve depth information [42]. Here, we study the effects of ego-movement on visual perception.

Regarding the effects of movement type on degradation artefacts, Petrescu et al. [43] showed that users’ head rotations can be used to drive an LOD simplification algorithm. Ellis and Chalmers [44] used dynamic configurations of FR regions scaled to the vestibular response. They used a 6DOF motion pod to simulate force typical of movement in a vehicle and found that this significantly affected sensitivity to different levels of degradations. More recently, Petrescu et al. [13] examined the effects of self and implied movement on a fixed FR implementation. They found that self-movement reduced sensitivity to more pronounced FR settings. Andersson et al. [45] showed that users did not report significant differences in FR artefacts in fast-paced VR games which require constant head movement compared to non-FR conditions. This supports the idea that head movement can be a reliable method to mask FR artefacts. Furthermore, Lisboa et al. [46] showed that rectangle-mapped FR can be enhanced during instances of implied user movement. Their results demonstrate that high-velocity movements through VR environments allow for a significant increase in the severity of their FR method when compared to stationary users. It is important to note that peripheral vision is widely attributed to motion detection [47]. motion detection [48] introduced a new method that compensates for motion artefacts in the periphery caused by FR implementations. This was achieved by superimposing motion cues at different energy values in the periphery to correct for the loss in motion sensitivity brought about by FR. Stimuli in our study are generally within the viewing frustum and not moving in the periphery, but exploring if the results in Tariq and Didyk [48] are consistent across different types of retinal motion is a promising avenue for future research.

Other applications of motion and rendering can be seen in Denes et al. [49], who created a vision model accounting for resolution and refresh rate that improved the visual quality of predictable and unpredictable motion. However, their study was limited to on-screen movement and did not consider other types of motion. VRS has also been used alongside models of visual acuity under movement to reduce rendering requirements [50, 51]. More recently, neural network super-resolution techniques accounting for temporal changes were used to great effect to optimise foveated graphics in VR [52].

In the current study, we build upon the research presented in Krajancich et al. [16], Petrescu et al. [13], Malpica et al. [8], and Ellis and Chalmers [44] to investigate the influence of attention-modulating tasks during movement on the artefacts induced by VRS foveated graphics. We also explore if different types of visual behaviours may affect sensitivity to FR in different ways. Our main focus is on understanding the extent to which different types of movement (active versus implied) and types of tasks (a simple task involving only fixation of targets versus a more demanding task involving fixation, discrimination between, and counting of appropriate targets) impact an individual’s ability to detect degradations in quality.

## Materials and Methods

### Overview

In this work, we explore the effects of task type and movement in two experiments each containing two separate parts. The tasks will be discussed in Section “Tasks”. Participants either moved by walking (Active Movement—AM condition) or were stationary and experienced a representation of movement (i.e. flying) through the environment (Implied Movement—IM condition). In order to investigate whether attentional mechanisms impact sensitivity to FR degradations, we devise a novel methodology inspired by the work in Huang and Dobkins [14], which is described in Section “Task”. As such, E1 (see Section “Experiment 1 (E1)—in Petrescu et al. [57]”) investigates the visual sensitivity to FR when users undertake visual search tasks with varying difficulties whilst moving. In E1 participants are allowed to employ an arbitrary type of visual search. In E2, we control the visual behaviour by instructing users to perform smooth tracking in order to detect the required stimuli (see Section “Experiment 2—Exploring Visual Behaviour”). For our FR method, we used NVIDIA VRS in its 4$\times$4 configuration (1/16 of the sampling rate of the non-degraded area, which is the most aggressive setting in our system) so that we obtain maximum benefits from our method and the down-sampling artefacts can be observed more clearly by the participants. We choose to use two foveation regions which we term HQ (High Quality, rendered at the native shading rate –  1$\times$1 pixel sampling) and LQ (Low Quality, rendered at VRS 4$\times$4). The dynamic foveated rendering algorithm we use was implemented by adapting HTC Vive code for foveated rendering [53] such that it functions with our current platform and XR SDK.

### Tasks

To explore how increased attention requirements—in this case, caused by task difficulty—can be studied using dynamic foveated rendering and in conjunction with different movement conditions and visual behaviours, we devise a novel method in VR. This method will be identical in both E1 and E2. The Rapid Serial Visual Presentation (RSVP) task from Huang and Dobkins [14] consists of a series of rapidly presented random letters sequentially (i.e. distractor letters) at a fixation point, with participants tasked with finding a target letter. Increasing the number of distractor letters is proportional to increased task difficulty and therefore the level of attention deployed by participants to complete the task. Krajancich et al. [16] adapted this method successfully to explore the effects of attentional load on foveated graphics.

**Fig. 1:**
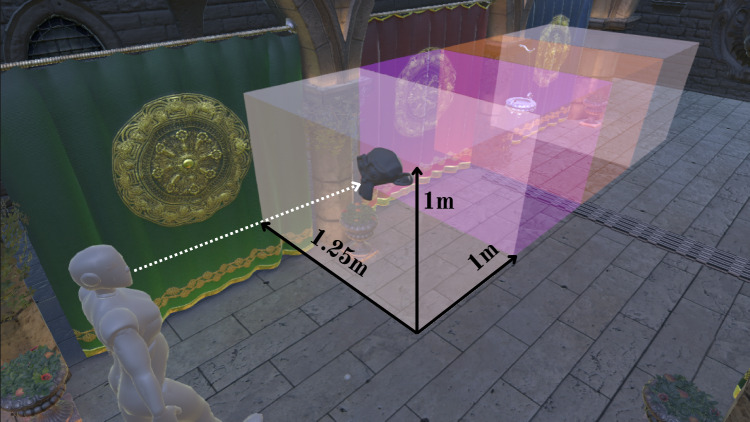
Illustrative example of the experimental set-up for a trial. Stimuli (here, Suzanne the monkey) appear in the designated spawn areas as the participant (here illustrated by a dummy model) is moving through the environment. The spawn areas were not visible to participants during the experiment and are illustrated here for clarity. The model is used to illustrate a hypothetical position of the participant undertaking a trial. When the participant entered the subsequent spawn area, all the stimuli from the previous area disappeared, even if they were not intercepted successfully

Inspired by this, we propose two tasks that will be used across E1 and E2: *Simple Fixations (SF)* and *fixation, discrimination, and counting*, which we will refer to as the *Monkey Finder (MF)* task. We divided the Sponza Scene corridor into four distinct spawn areas for task objects. Note that the y-axis value (i.e. vertical position) of the spawn volume is scaled to the height of the participant at the beginning of the experiment for consistent representation. In E1, across both tasks, participants move through the scene and are instructed to look at appearing objects. Objects appear sequentially before the participant enters each spawn zone so that items always appear within the FOV of the viewing frustum. When an object is intercepted by the gaze of the participant, it blinks once by changing colour and then disappears. Objects spawn randomly within the volume of the spawn area (1.25$\times$1$\times$1 ms). Each stimulus appears in the spawn area when the viewer is 0.75m away from it (see Fig. 1). This ensured that objects are always reasonably placed in the viewing frustum and prevents participants from having to perform ballistic movements to intercept the stimuli.

In E2, the same experimental paradigm is used with the addition of a guiding sphere that is moving towards the position of the appearing objects. As such, the visual behaviour does not involve searching for objects in this case. The same tasks (MF and SF) and movement conditions (AM and IM) were explored in E2. This permitted us to explore if the results in E1 are consistent during instances of tracking.

For both experiments, each corresponding trial in each task condition was allocated the same positional values for task objects at runtime (e.g. items in trial 1 in MF had the same positions as in trial 1 in SF). This gave us confidence that the participants performed roughly the same eye fixations (E1) or followed the same visual path (E2) in both tasks. At the end of each SF and MF trial participants were asked if they noticed a degradation. To provide a reference, participants always saw the undegraded scene (i.e. native shading, VRS1$\times$1) prior to beginning AM or IM.

#### Simple Fixations (SF)

**Fig. 2:**
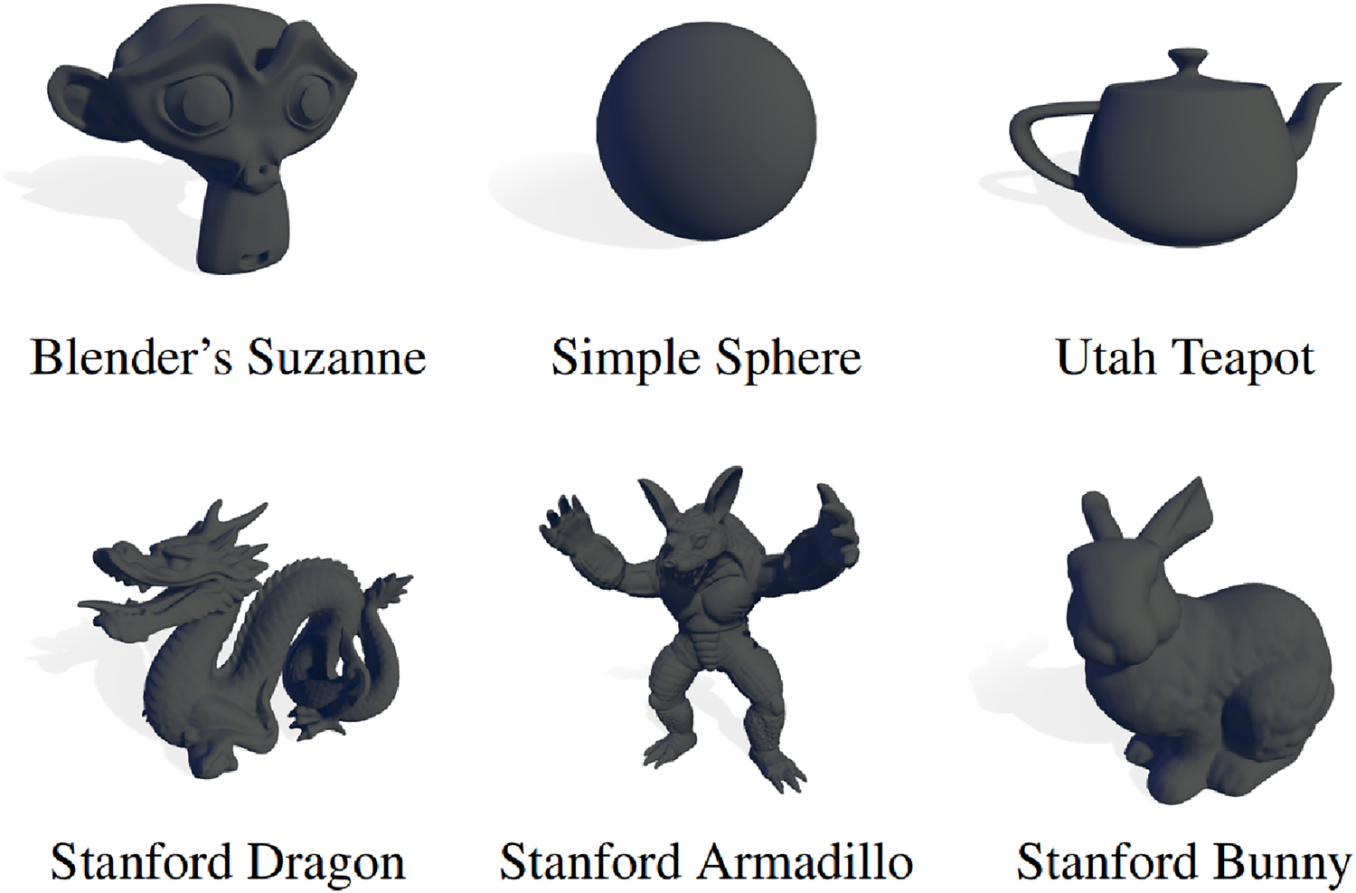
Computer graphics models used for MF and SF tasks, including Blender’s Suzanne, a Simple Sphere, the Utah Teapot, and the Stanford Dragon, Armadillo, and Bunny. Source: Petrescu et al [57]

In the SF condition, participants move through the environment and are presented with spherical objects (top-middle in Fig. 2). Participants are instructed to fixate on spheres (in E1) or track the guiding object towards the location of where the sphere will appear (in E2) as they progress through the environment. Note, in both tasks each spawned object was textureless and assigned a random colour (in the RGB colour model).

#### Monkey Finder (MF)

In this task, participants move through the environment (AM and IM) like in SF. In E1, they are instructed to fixate on items that appear sequentially and to count the occurrences of a target object (here, *Suzanne, the Blender Monkey*, top-left in Fig. 2). Note that in E2, the task was the same but they followed the guiding sphere towards the appearing objects. Compared to SF, the objects that appear on the screen can now be any of the models presented in Fig. 2. The probability of a monkey appearing was set to 33%, compared to 13.2% for the distractor objects. This was to ensure participants were actively counting and staying engaged with the task. At the end of the trial, participants were asked how many monkeys they spotted (between 0-4) in addition to the degradation question.

### Stimuli

**Fig. 3:**
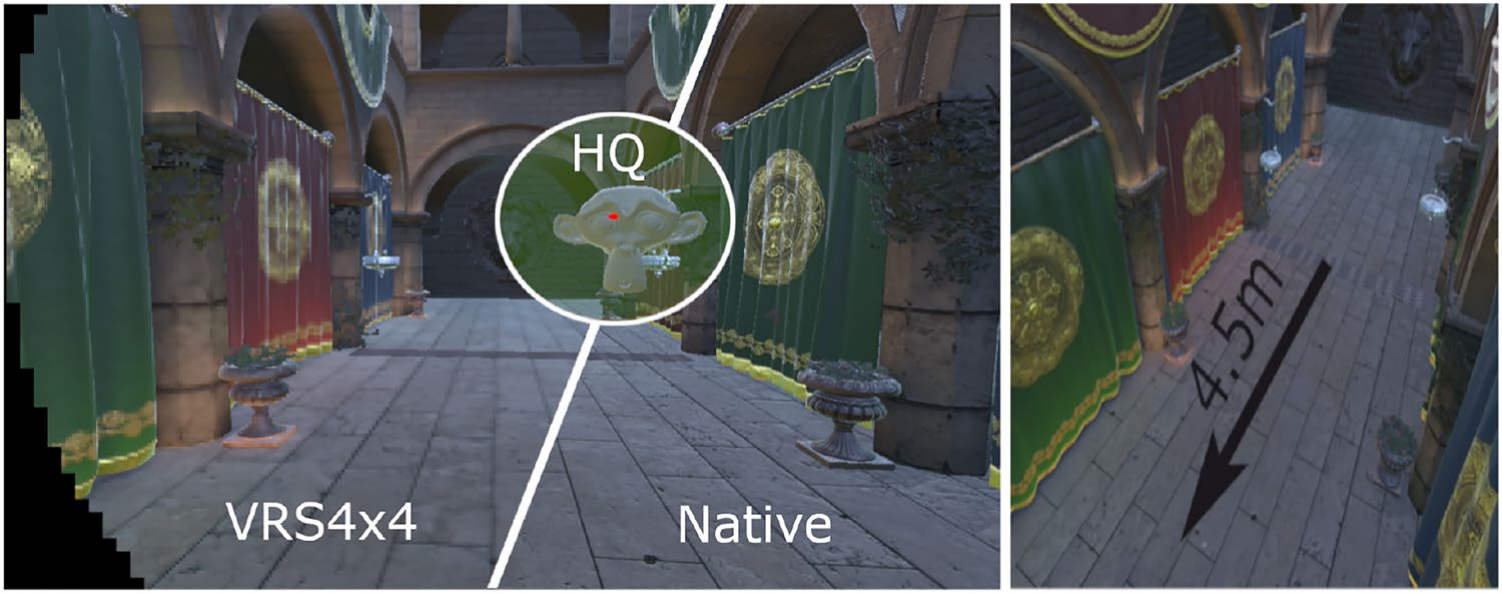
Left: representation of VRS4$\times$4 shading reduction; in green - foveated region (HQ); the red dot at the centre of the foveated region represents the eye-tracked focal point. The black margins show the algorithm culling everything outside the peripheral FOV. Towards right: a comparison to native shading (VRS1$\times$1). Right: the path participants walked scaled to the dimension of the facility. Source: Petrescu et al. [57]

The popular Crytek Sponza scene was adapted and rendered in Unity (Universal Render Pipeline). We scaled it to match the dimensions of the VR2 facility. The facility has a length of 5.5 m; our path was capped at 4.5 m (Fig. 3, right). We turned all the anti-aliasing and lighting probes provided by Unity to restrict participants’ judgements only to VRS degradations.

In E1, we added a semitransparent guiding sphere that occupied 2° of visual angle in front of the participants and served as a guide for the speed they had to achieve. The sphere accelerated to 0.75 m/s in 0.25 s in order to mimic the acceleration of a human initiating walking. This speed was chosen to match typical walking speeds in VR, which are slower than real locomotion [54]. When the sphere reached its peak velocity, the VRS degradation was triggered.

In E2, participants were also tasked with keeping their gaze fixated on the guiding sphere. The sphere then moved towards the target objects in MF and SF (see Fig. 6). This was to ensure that the participants were performing a tracking task and not a visual search. The velocity of the sphere was lowered to 0.5m/s in E2. This speed was chosen in order to fit the range of velocities studied in Jindal et al. [50] for motion-driven VRS optimisation for VR, but also to ensure reliable tracking and limit catch-up saccades [55]. Note that the sphere would only carry on its movement if the participant was positioned at least 1 m behind it to maintain a retinal image of 2° like in E1; the guiding object waited if the participant did not match its speed to ensure roughly the same proportion of the visual field is covered.

If the participant’s gaze was directed towards the sphere in E2, the object would have a blue-ish colour (see Fig. 6). The colour would change to orange-ish if the gaze was directed to a different part of the scene to draw back attention to the guiding object and maintain the intended visual behaviour. The colours were chosen according to the ‘Universal Color Design Pallete’ in order to accommodate individuals with colour blindness [56].

Before the experiments, participants were asked to complete practice trials. Firstly, they were shown what the VRS degradation looked like at different configurations. Afterwards, participants were also asked to complete SF or MF trials until they felt comfortable with the task. This ensured that the difficulty was consistent across the participants. In each trial, participants were informed whether they were performing SF or MF (interleaved) and then initiated movement. At the end of the trial, participants were presented with an answer screen where they were asked if they noticed any degradation. If they had performed the MF task they were asked how many monkeys they counted and whether they noticed any degradation by pressing either the *yes* or *no* button. In the walking condition, they were asked to rotate and press the trigger on the controller to start the next trial. In the passive condition, they simply pressed the trigger to initiate the next trial.

### General Design

For both E1 and E2, we used a 2$\times$2 fully factorial within-subjects design for a total of four conditions per experiment. Our independent variables were: Type of Movement (AM versus IM) and Task Type (SF versus MF). We compared performance on our dependent variable (i.e. the probability of participants not noticing the smaller HQ diameters) between conditions. For each trial, the diameter of the HQ region (Fig. 3, left) was varied using an adaptive staircase (Kestsen staircase). The diameter varied in line with two interleaved staircases for each of the four experimental blocks (8 staircases in total). Per condition, one staircase started with a large diameter (i.e. HQ = 90°), and the other started with zero diameter (i.e. full degradation, HQ = 0°). Note that the large diameter does not cover the full FOV of the HMD used. Based on the findings in Petrescu et al. [13], an HQ of 90° is suitable for this kind of experiment. Moreover, a smaller upper limit means that more data will be collected in the area of interest (i.e. where the two interleaved staircases will overlap). For more information about the Kesten staircase, refer to Section“ Kesten Staircase”. Each staircase comprised of 40 trials. Catch trials were added throughout each part of E1 and E2 in order to ensure participant engagement. In a catch trial, we presented a VRS4$\times$4 degradation where HQ diameter = 0°, and all of the spawned objects were spheres that appeared in the centre of the visual field. Therefore, if a participant performs the task as intended, they should always see the VRS degradation in a catch trial. We presented eight catch trials per experimental condition in a randomised order, resulting in a total of 336 trials per participant across the four conditions. To minimise fatigue, the experiments were both run in two sessions; one in which participants completed the trials by performing AM (with SF and MF) by producing self-induced surge motion (i.e. forward walking), and one in which participants were stationary and were moved through the scene (i.e. the IM condition with SF and MF). The order of the sessions was also randomised across participants. As such, half of the participants undertook the AM condition first (and IM afterwards), and half the IM condition first (and AM afterwards) to prevent learning effects. This was consistent across E1 and E2.

### Kesten Staircase

The *Kesten Adaptive Staircase*, or *Accelerated Stochastic Approximation* is an algorithm widely used in psychophysics to systematically vary the magnitude of the studied stimuli across the whole intended experimental range. It offers the advantage of converging more rapidly towards a threshold when compared to many other adaptive staircase procedures [58]. This also ensures that a minimum number of trials are required for each participant, addressing potential fatigue issues. This is especially important for VR, as longer experiments are associated with a decrease in task performance and increased eye strain [59, 60]. Note that a limitation of the Kesten staircase is that if the participants incorrectly identify the change in stimulus (in our case degradation) during the initial trials, there is a risk that the staircase will not properly converge. Consequently, this would prevent data from being fitted on a psychometric function (henceforth PF, see Section Experiment 1 (E1)—in Petrescu et al. [57]). The convergence points for the staircases in E1 and E2 were two symmetrical values around the 75% performance threshold for VRS degradation (i.e. the diameter at which a participant has a 75% chance of not detecting the degradation). This means they were three times more likely not to notice the change than to notice it. By setting the convergence point around 75%, we ensure more data will be collected around the range of interest.

1 $$\begin{aligned} diam(HQ) = x_{n+1} = x_{n} - \frac{step}{2 + m_{revs}}\left( resp_{n} - \Phi \right) , n>2 \end{aligned}$$

Equation 1 represents the accelerated stochastic approximation. For the first two trials, this does not differ from the standard stochastic staircase procedure [58] (i.e. the reversals in the response category are not accounted for). The value of $diam(HQ)$ represents the diameter of the HQ region that is manipulated in each trial using the result obtained from the Kesten procedure; as such, the diameter of the HQ region is derived from the answer the participant gave in the previous trial. The variable $m_{revs}$ is a cumulative count of the number of times participants reversed their responses. The variable $resp_{n}$ is a binary value (*yes = 0* or *no = 1* in our case) representing the answer the participant gave about whether they noticed the degradation for the $n^{th}$ (i.e. the previous) trial in the staircase. We use two interleaved staircases. One ascends towards the threshold from HQ = 0° upwards, and one descends from HQ = 90° downwards. This generates more data around the performance point of interest (here, 75%). We chose $x_0 = 0, \Phi = 0.875$ - ascending; $x_0 = 0.9, \Phi = 0.625$ - descending. Note that the convergence points are set to ensure that the staircases will be partially overlapping in order to prevent gaps in the data. The $step$ value was set at 0.45. This value controls the initial step size, which is reduced as a function of the number of reversals.

### Apparatus

The data for this experiment were collected in the Virtual Reality Research (VR2) facility at the University of Manchester.^1^ The Oculus Quest Pro headset with eye-tracking enabled was used. The HMD’s resolution is 1800$\times$1920px per eye with a rendered horizontal FOV of 108°. As the study involved actively moving through the environment, we used the Oculus Air Link for mobility. The data was transferred at 90Hz with the rendered FPS capped at 90 on our desktop configuration (Intel i7-7700K CPU and NVIDIA RTX 3080 Ti GPU). Empirical work suggests that the Unity SDK for the Oculus Quest Pro caps the eye-tracking frequency at 72 Hz [61]. This might introduce noticeable artefacts for saccades performed over large distances. However, piloting the experiment did not raise any major concerns with regard to inaccurate tracking. HMDs tend to vary in effective resolution across the display because of the optical properties of the lenses. Beams et al. [62] showed that the effective resolution in VR HMDs decreases rapidly for off-axes angles (i.e. towards the visual periphery). This effect was however reported for headsets with fresnel-type lenses. The Quest Pro used in our study has pancake lenses which improve optical quality edge-to-edge [63]. Whilst this could potentially impact the visibility of FR/VRS artefacts, the purpose of this experiment is not to report a specific compression or a novel FR method, but to study the interaction between the experimental conditions. The equipment used in E1 and E2 was the same across all participants, meaning any differences in outcome can be attributed to the experimental manipulations.

The experiment was coded in the *Unity* game engine (Version 2022.3.4f1) and verbose data about each trial was collected using the Unity Experiment Framework (UXF), which was designed for the development and control of psychophysical studies [64].

### Experimental Procedure

For both E1 and E2, participants completed the experiment in two separate sessions. In one of the sessions they performed AM (SF and MF tasks interleaved) and in the other, IM (SF and MF tasks interleaved). Participants were randomly assigned either AM or IM as their first experimental condition to prevent order and learning effects. At the beginning of each session, we calibrated the eye-tracking with the Oculus application, with additional manual calibration performed if required. After a countdown and a text message informing the participants that they were doing MF or SF, they initiated movement. For E1, in the AM condition, they were instructed to match the speed of the fixation sphere, with the sphere changing colour if they were not fast enough. For E2, they had to match the speed of the sphere and also keep their gaze directed towards it as the sphere would change colours otherwise. At the end of an AM trial, participants answered the task-specific question and then were asked to rotate 180° until aligned with a straight line that appeared in the VR environment. At the beginning of each trial, the scene was rotated to match the midsaggital plane of the participant in order to account for small deviations in participant orientations and to present a consistent representation of the Sponza environment. In the IM trials, participants were stationary, but we aligned the scene in each trial to account for small movements.

### Pre-screening

For both E1 and E2, participants were allowed time to acclimatise to the VR paradigm and the VE. In order to confirm task comprehension, pre-experimental practice trials were run in each condition until participants verbally confirmed their comfort with the task. Additionally, whilst stationary, we presented VRS degradations with HQ = 0° (full degradation) and HQ = 5° (severe degradation) and asked them to compare it to the scene rendered at 1$\times$1 shading (native shading, no degradation). All participants in both E1 and E2 were able to reliably detect these differences.

## Experiment 1 (E1)—in Petrescu et al. [57]

We formulated three hypotheses for E1 which were pre-registered with the Open Science Framework (OSF) at https://osf.io/wkptz. Note that we retrospectively changed the pre-registered term ‘cognitive load’ to ‘attentional load’ in this paper. We do not deviate from our pre-registered analysis plans.

- H1: Virtual Reality users engaged in tasks that require a higher attentional load will tolerate higher levels of degradation in a foveated system compared to those users not engaged in high attentional load tasks.
- H2: Users engaged in Active Movement will tolerate a significantly higher level of degradation compared to users engaged in Implied Movement.
- H3: Users who are both walking and performing higher attentional load tasks will tolerate greater levels of degradation compared to users subject to all other conditions/combinations of conditions.

### Participants

Data were collected from 15 participants (12 naive to the study, and three involved with designing the experiment). All participants were staff members at the University of Manchester. Data from two participants were discarded because their responses did not converge and it was therefore not possible to fit PFs to their data, and from one because they did not complete both parts of the experiment. The study was approved by the Ethics Committee of The University of Manchester, Division of Neuroscience and Experimental Psychology. Written consent was collected from all participants. The AM session lasted $\approx$ 40 min and the IM $\approx$ 25 min. The effective time of the experiment was between 60 and 70 min per participant across two sessions on separate days with breaks offered every 20 min to prevent fatigue and preserve data quality. Eye-tracking data was collected from all participants. We added an additional eye-tracking calibration test at the beginning of the experiment to make sure the Quest Pro calibration was accurate.

### Validation

The catch trial results show that, across conditions, participants could correctly spot VRS degradations. Eight participants correctly identified the degradation across all catch trials, and four only responded incorrectly to 1 catch trial out of a total of 16. To make sure the participants completed the tasks as intended, we recorded the number of objects in the scene they gazed at over all trials. Across participants, the average number of objects that were gazed at is 3.79 out of 4 (total number of objects spawned per trial). This gave us confidence that movement did not prevent participants from performing the task as instructed. Furthermore, we recorded the number of correct answers in the MF task. There was no significant difference between the proportion of correct answers in AM (93%) and IM (94%) MF conditions; $t(22.14) = 0.30, p = 0.75$, which provides evidence that the task was performed as instructed in both conditions.

### Psychometric Functions (PF)

**Fig. 4:**
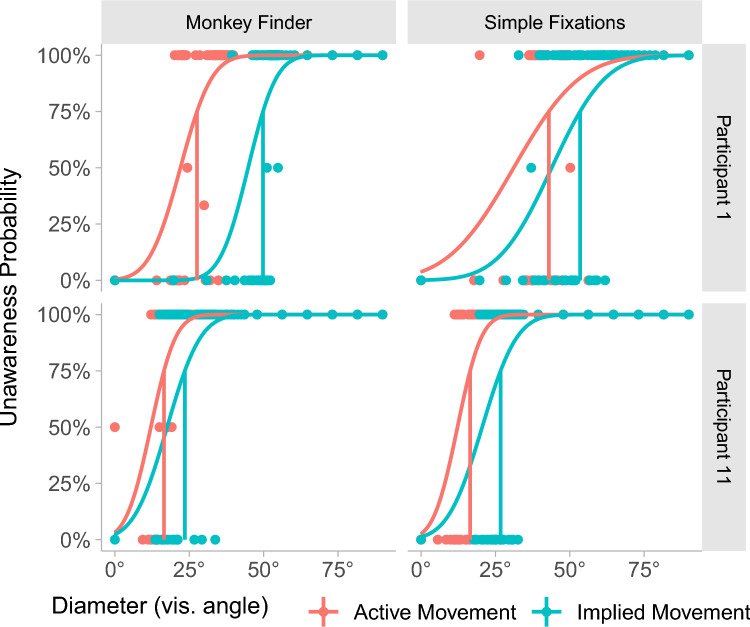
Example psychometric function fits for two participants in E1. Each of the MF and SF tasks are plotted separately, with unawareness probability on the *y* axis, and the diameter of the foveated region in degrees of visual angle on the *x* axis. Psychometric functions are plotted separately in each task condition for active and implied movements. Source: Petrescu et al. [57]

We fit the binary response data in our experiment using a PF fitting approach. PFs model how perceptual responses relate to variations in a physical stimulus; here, the diameter of the HQ region, which varied in line with the Kesten staircases. This physical measure is then mapped to the perceptual response. This represents the probability of the participant not noticing the shading reduction at each foveation level. We used a cumulative Gaussian PF with two free parameters—mean and standard deviation. The cumulative Gaussian was chosen due to its straightforward interpretation over other psychophysical models. Example fits can be seen in Fig. 4.

The data were fit using the *quickpsy* package (Linares and Lpez-Moliner [65]) in R (Version 4.2.3, R Core Team [66]). This package provides functions that fit the PF to binary response data using a maximum-likelihood approach. From this fit, we recovered the HQ diameter at which participants had a 75% probability of not noticing the degradation. This represents the point at which participants are three times more likely to not notice the degradation than to notice it. We refer to this point as *maximum tolerated diameter* (MTD). This nomenclature will be consistent in E1 and E2. Note that in this experiment, lower MTD values for the HQ region mean more scope for optimisation (see Table 1).

### Results

**Fig. 5:**
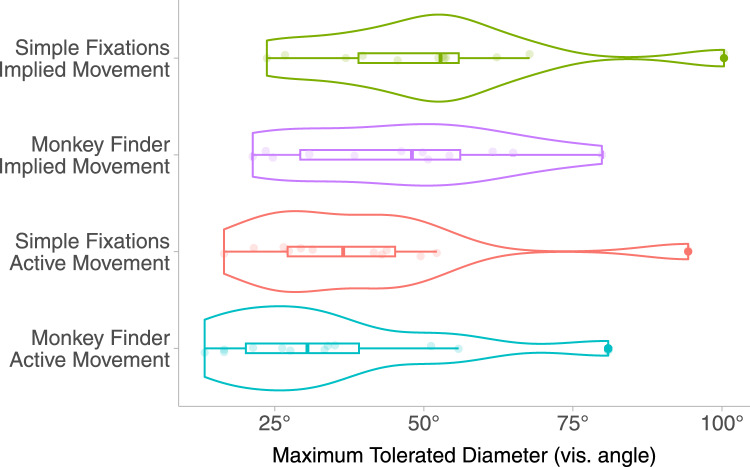
Violin plots showing descriptive statistics of all MTD points in E1 across all combinations of conditions (left). Source: Petrescu et al. [57]

Having satisfied the Kolmogorov-Smirnov test for normality, we use ANOVA to analyse our MTD data. We plot the mean value of the MTD points (75% likely to notice a degradation) for our dataset in Fig. 5. Smaller MTD values indicate an increased tolerance for a larger degraded area, therefore creating more scope for optimisation. The mean thresholds span from 34.3° to 51.3° in all conditions (see Table 2). This shows that even in a very aggressive VRS 4$\times$4 configuration, there is significant scope for the reduction of shading quality. Figure 5 suggests that both MF and AM manipulations lead to a greater potential for more severe foveated rendering over SF and IM conditions respectively. In support of these observations, significant main effects of these factors resulted from a 2$\times$2 repeated measures ANOVA (Table 1). The $F-value$ in ANOVA measurements represents the ratio of variance between the means of the experimental groups. These results provide evidence that the MTD is modulated by a main effect of task ($F(1,11) = 7.00, p = 0.02$) and a main effect of Type of Movement ($F(1,11) = 14.55, p = 0.003$). However, we do not find a significant interaction between the two main effects ($F(1,11) = 0.03, p = 0.87$), suggesting that their contributions are additive.

**Table 1:** Descriptive statistics of the ANOVA analysis in E1—source: Petrescu et al. [57]

Conditions	F stat	*p*-value	$\eta _{p}^{2}$
Task	7.00	0.02*	0.39
Type of Movement	14.55	0.003*	0.57
TypeMov * Task	0.03	0.87	0.003

**Table 2:** Mean MTD (in degrees) values in E1—Source: Petrescu et al. [57]

	AM	IM
Monkey Finder	$\hbox {34.3}^\circ$	$\hbox {45.4}^\circ$
Simple Fixations	$\hbox {39.7}^\circ$	$\hbox {51.3}^\circ$

The results obtained here support our hypotheses. We showed that:

- A task that requires more attention causes participants to be significantly less likely to notice degradations (**H1**).
- When performing active movement, participants are willing to tolerate a significantly smaller HQ region (**H2**).
- The mean MDT value obtained for the MF-AM task is lower than for all other conditions, indicating that the combination of active movement and the presence of a high attentional load task results in lower MTD values (**H3**). The lack of a significant interaction between the main factors indicates that the effect is additive in nature regarding participants’ tolerance to foveation.

## Experiment 2—Exploring Visual Behaviour

**Fig. 6:**
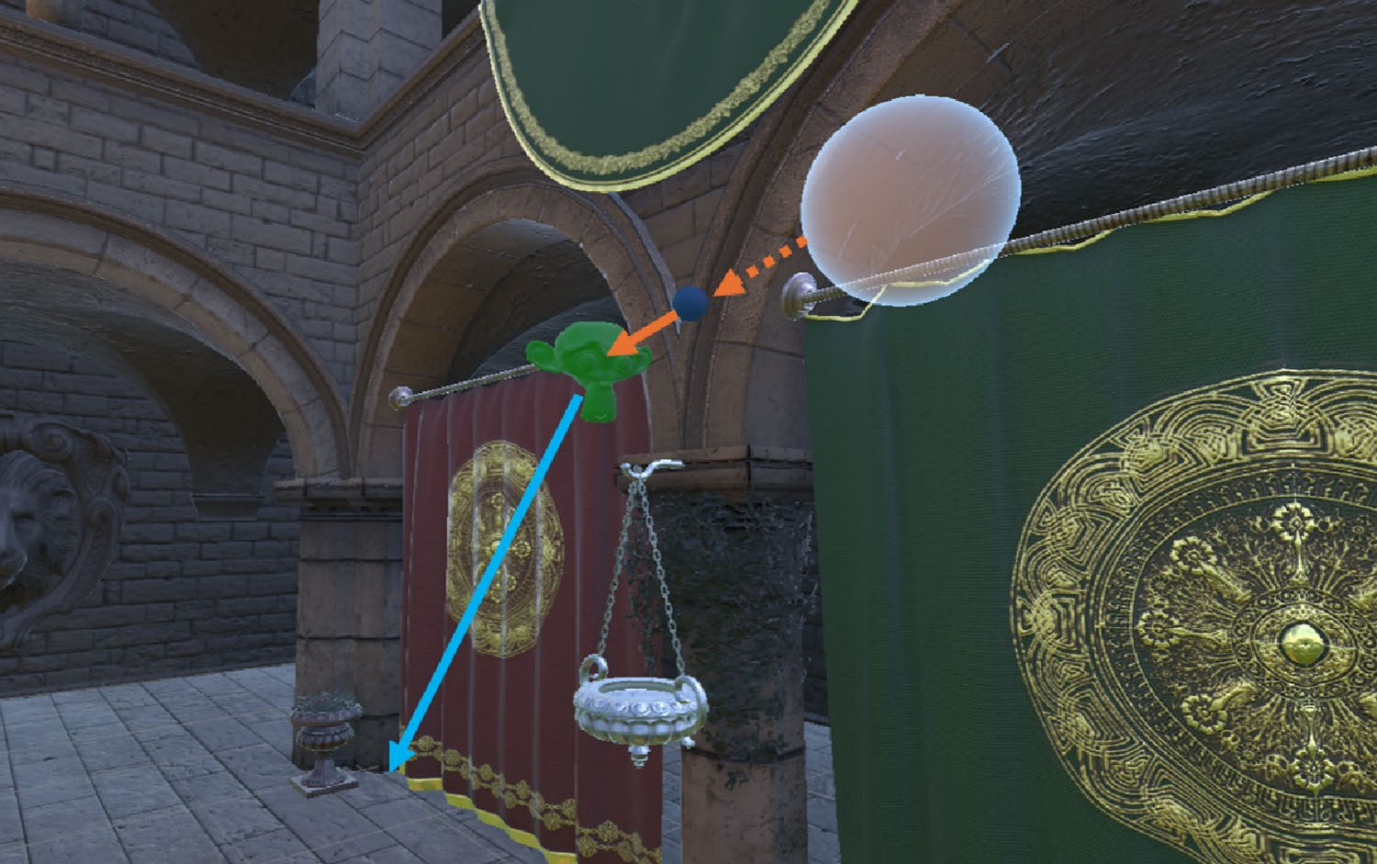
Example MF trial in E2. An illustrative representation of the guiding sphere (blue) being tracked. The orange arrow (dotted) represents the path already undertaken by the sphere and the solid arrow represents the remaining movement. The blue arrow represents a hypothetical direction of the sphere after the participant has looked at the task object and is within 1 m of the sphere. The transparent white sphere is illustrative, and represents an object the participant has looked at before the sphere initiates movement

VR users are often required to perform tasks which include visual tracking or pursuit (refer to Fig. 6). Typical VE behaviours that involve visual tracking might be: pursuing enemies, following characters or objects, or tracking projectiles. Because of this, it is important to understand if the findings in E1 are consistent during the deployment of different visual behaviours typical in a VE. The same tasks (MF and SF) were used in order to contrast the findings with E1 (see Section “Task”). For the implementation of the guiding sphere, see Section “Stimuli”. We formulated three hypotheses for E2:

- H1: Virtual Reality user who perform tracking and are engaged in complex tasks which require the deployment of higher attentional loads will tolerate higher levels of degradation in a foveated system compared to those users not engaged in high cognitive load tasks.
- H2: Users performing tracking and Active Movement will tolerate a significantly higher level of degradation compared to users engaged in Implied Movement and tracking.
- H3: Users who are tracking a moving fixation and are involved in both walking and performing higher attentional load tasks will tolerate greater levels of degradation compared to users subject to all other conditions/combinations of conditions.

### Participants

We recruited 10 participants for E2 (eight naive to the study, and two involved with designing the experiment). The number is lower than E1, but due to the nature of psychophysical studies with high numbers of trials, we were confident the data obtained is representative [67]. All participants were staff members at the University of Manchester. Data from three participants were discarded: one of the participants provided data that did not converge on the staircase and it was therefore not possible to fit the PFs reliably; one participant did not complete both parts of the experiment; the final excluded participant only gazed at the guiding sphere 38% of the trial time in the IM condition and 49.9% in the AM condition. Due to the recorded values, we were not confident the participant executed the task as instructed and excluded them. The study was approved by the Ethics Committee of The University of Manchester, Division of Neuroscience and Experimental Psychology. The sessions were marginally longer than in E1 due to the slower speed of the guiding sphere. Consequently, the AM session lasted $\approx$ 45 min and the IM $\approx$ 30 min. The effective time of the experiment was between 65-80 min per participant over two separate sessions on different days with breaks offered every 20 min in order to prevent experimental fatigue.

### Validation

The number of catch trials and experimental trials was the same as in E1. The catch trial results show that, across conditions, participants could correctly spot VRS degradations. Most participants reliably identified the degradation across all catch trials, with three having correct response rates around 90%. Similar to E1, we recorded if participants looked at all the objects in the scene over all trials. Across included participants, the average number of objects that were gazed at is 3.799 out of 4. This value is almost identical to that recorded in E1. Therefore, we maintain confidence that participants performed the task as instructed. We also recorded the percentage of frames in which the guiding sphere was correctly tracked. The mean percentage across participants was $\approx$ 75% (with one participant excluded). Additionally, we recorded the number of correct answers in the MF task. There was no significant difference between the proportion of correct answers in AM (82.9%) and IM (92.3%) MF conditions $t(7.23) = -1.46, p = 0.19$. The addition of the visual tracking behaviour did not significantly increase the difficulty of the MF task to the point where participants could not reliably complete the experiment.

### Results

**Fig. 7:**
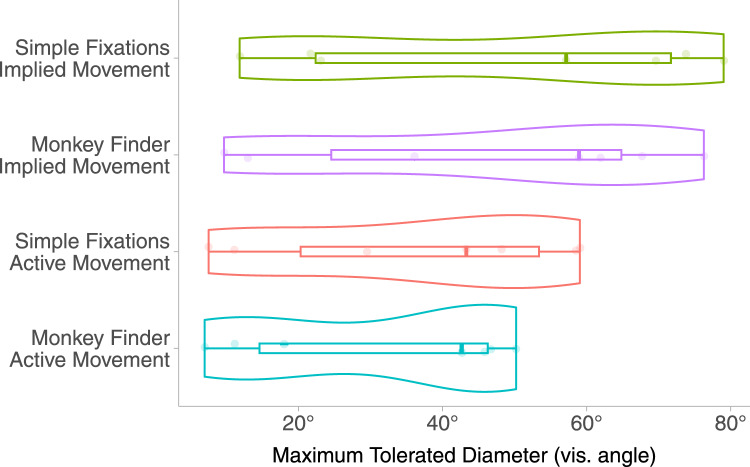
Violin plots showing descriptive statistics of all MTD points in E2 across all combinations of conditions (left)

The framework used to fit the data to PFs was identical to E1 (presented in Section “Psychometric Functions (PF)”) (see Table 3). The MTD values recovered satisfied the Kolmogorov-Smirnov test for normality in all conditions. The MTD values for E2 are plotted in Fig. 7. We observe that MTD values span from 31.7° to 48.8° in all conditions (see Table 4). These values are lower than in E1 and suggest that different visual behaviours have the potential to modulate sensitivity to FR artefacts. In support of these observations, we find a significant main effect following the analysis of a 2$\times$2 repeated measures ANOVA (Table 3). The MTD is mainly modulated by the main effect of Type of Movement ($F(1,7) = 10.77, p = 0.02$). Interestingly, there is no main effect of Task Type in E2 ($F(1,7) = 2.40, p = 0.17$). Consistent with E1, there is no significant interaction between the two main effects ($F(1,7) = 0.66, p = 0.45$). These results suggest that the addition of a different visual behaviour involving a tracking task diminishes the effect of Task Type. This effect is further explained by looking at the effect size of the Task effect in both experiments. We further contrast the effect size values (represented by $\eta _{p}^{2}$ in Tables 1 and 3) between the conditions in E1 and E2. $\eta _{p}^{2}$ is a measure of effect size quantifying the impact of a certain independent variable on the dependent variable. It ranges from 0 (no impact) to 1 (large impact). By comparing effect size in E1 ($\eta _{p}^{2}$ = 0.39) and E2 ($\eta _{p}^{2}$ = 0.29), the change in visual behaviour seems to diminish the effect of the attentional load required for different tasks.

**Table 3:** Descriptive statistics of the ANOVA analysis in E2

Conditions	F stat	*p*-value	$\eta _{p}^{2}$
Task	2.40	0.17	0.29
Type of Movement	10.77	0.02 *	0.64
TypeMov * Task	0.66	0.45	0.10

The notation “TypeMov * Task” represents the interaction between the independent variables

**Table 4:** MTD values in E2

	AM	IM
Monkey Finder	$\hbox {31.7}^\circ$	$\hbox {46.2}^\circ$
Simple Fixations	$\hbox {36.8}^\circ$	$\hbox {48.0}^\circ$

To summarise, the results presented here demonstrate that:

- A task that requires more attention during visual tracking does not cause the participant to notice significantly higher FR optimisations **(H1)**.
- When performing active movement, participants are willing to tolerate a significantly smaller HQ region (thus supporting **H2**).
- The mean MDT value obtained for the MF-AM task is lower than for all other conditions, indicating that the combination of active movement and high attentional load tasks is additive in nature regardless of the visual behaviour (**H3**).

## Discussion

The findings from E1 and E2 have potential implications for the design of FR algorithms and the enhancement of models of visual perception. During typical VR usage, users are often engaged in tasks requiring visual search or tracking (e.g. finding items, discriminating between allies and other characters, or tracking and aiming at targets). Movement in VR arcades is becoming increasingly popular, especially that which is self-induced [68]. Along with the recent release of MR devices, it is expected that stationary at-home immersive experiences are rapidly being converted to more dynamic scenarios. Furthermore, immersive environments are becoming more common for applications in medical [69] and military [70] training, exposure therapies and anxiety interventions [71, 72], and therapy for motor and neurological disorders [73, 74]. Moving away from the stationary VR paradigm means that understanding how different types of movement affect optimisation methods such as FR will be critical in future systems. The novel methodology introduced in this work allows us to explore how different combinations of types of movement, tasks, and visual behaviours interact and affect the severity to which FR can be used. All these are important aspects of immersive media that have received insufficient attention from the graphics community in the past. The method used here allows for the analysis of various combinations of the aforementioned conditions during instances of forward surge movement. We provide a fully dynamic VRS-based FR implementation for this study. Whilst we do report potential savings, we do not claim a fully functional foveated algorithm as this study is exploratory in nature.

The findings in Petrescu et al. [13] show that during instances of AM (also forward surge), an HQ region $\approx$ 38.3% of the FOV of the headset was required (note that the same HMD was used in the VRS4$\times$4 configuration). In their method, participants kept a stationary gaze towards the centre of the screen in a fixed FR method. In E1 and E2, we used a dynamic FR method and suggest that when accounting for Task Type and Type of Movement, the HQ region only needs to occupy 31.7% of the rendered FOV during visual search and 29.3% respectively during tracking (E2). Ellis and Chalmers [44] created a similar model that scaled the HQ region to the hypothetical force experienced by the vestibular system. Note that they used a 6DOF motion pod to simulate movement and have control over the vestibular response. They created a dynamic map of ego-movement that guided sampling and found an HQ region of 65% of the FOV. It is important to mention, however, that they used a different downsampling method of a much older renderer, rather than a VRS-like solution, which means that a direct comparison is not completely possible. In our case, information about the vestibular response could be derived from movement parameters and could be used to improve the system.

Malpica et al. [8] provided evidence that task type influences visual behaviour. In their study, during visual search in VR (a similar paradigm to ours in E1), participants exhibited significant differences in visual behaviour resulting in larger saccades and shorter fixations during scene analysis. Therefore, visual artefacts generated by VRS could be masked by mechanisms of saccadic suppression during visual search [12]. However, in E2, we show that there is more scope for degradation during visual tracking when compared to search. Therefore, it seems that the visual behaviour itself could be masking more severe FR artefacts. Moreover, in the study in Malpica et al. [8], participants are moved through the environment (which we term implied movement) at a steady pace and do not produce self-induced movements, while we show that AM significantly modulates sensitivity to FR. These findings are supported by those in Angelaki and Hess [41], which suggest that during instances of self-movement, peripheral acuity is compromised in order to limit retinal slip around the focal point. Lisboa et al. [46] suggested that increasing the velocity of the user in instances of implied movement (i.e. participants flying through the environment) significantly reduces their ability to detect peripheral degradations caused by FR; the velocities in that work range up to 200 km/h. The velocities used here in E1 and E2 are typical of those which users normally perform during VR usage. However, the findings from Lisboa et al. [46] motivate further work in which multiple velocities for AM are explored using a motion platform. It is important to note that during AM, vestibular, visual, proprioceptive, and efference copy systems are presented with consistent and competing inputs which interact in complex ways [75]. This interplay might affect the way users perceive foveated rendering degradations. In support of this claim, there is evidence that retinal motion is minimised during instances of forward surge in order to preserve depth information [42] or prevent retinal slip Angelaki and Hess [41], which could resolve the spatiotemporal aliasing caused by VRS.

Additionally, we provide evidence that the addition of difficult tasks (which require more attention) represents an untapped resource with regard to maximising foveation benefits. This is supported by the findings in Krajancich et al. [16], which show that tasks which require increased attentional load result in lower sensitivity to peripheral loss of detail. We observe a similar pattern of results in E1, where the addition of a more difficult task (Monkey Finder) significantly decreases sensitivity to FR. Montagna et al. [15] explain that during the deployment of covert attention, peripheral acuity is compromised in order to recover more detail around the point of focus. In E2, the addition of a different visual behaviour seems to saturate the effects of task type. We believe that this discrepancy could be attributed to the increased attentional load participants experience during tracking which causes noise across the Task Type condition.

### Limitations and Future Work

Firstly, the eye tracker frequency of 72Hz may cause noticeable

artefacts. Whilst there is evidence that critical fusion frequency, or the frequency at which a flickering stimulus can be perceived as continuous, is between 50-90Hz, it may be as high as 500 Hz [76]. We observed that in some cases, eye fixation over a long distance caused delays in the HQ region being updated which sometimes resulted in inaccurate HQ positions. Raw gaze data were not collected during the experiment because the stimuli locations were the same in corresponding trials from different conditions; this ensured participants were performing roughly the same eye movements. Second, the sudden activation of the VRS degradation might have been used as a strategy to detect the degradation rather than the downsampling. Due to the popping effect caused by the VRS activation, a visible flicker was briefly present. This will be addressed in future work and we believe a dynamic scaling such as the one employed by Ellis and Chalmers [44] could be used to address this issue. The study is also limited by the lack of variety in the environment. Exploring if different scenes (e.g. natural, artificial, checkered patterns) significantly modulate the results presented here would be necessary to claim these effects are universal. However, this would have caused a combinatorial increase in the total trial number which was not feasible here. Finally, most FR algorithms involve the use of at least three foveation and/or blending layers in order to address foveal, medial, and peripheral vision. We only used two to limit the total experimental time. It is important to mention that unlike the RSVP task [14] found in Krajancich et al. [16], the SF and MF tasks do not scale in a convenient manner. This prevented us from extracting more data points across levels of attentional load. Nevertheless, given that the experiments were already very long, adding extra dimensions in order to be able to fit a model in a within-participants study would be time-consuming and expensive to run.

The results of exploring the coarse perceptual effects of attention, movement, and visual behaviours on visual acuity in E1 and E2 raise important questions. We show that the effects of movement in E1 seem to remain consistent in E2. This supports the idea that surge AM can be reliably used to optimise FR regardless of the visual behaviour. The significant effect of Task Type we found in E1 seems to be diminished during visual search. The complex array of processes involved in tracking (e.g. retinal feedback for prediction, appropriate target selection etc.) has been shown to increase cognitive load [77]. In addition, there is evidence that the accuracy of tracking and pursuit are affected by increased cognitive load [78]. Therefore, the results in E2 could be explained both by the extra processing required by the tracking of objects and the increased attentional load required by the task. Note that the number of participants was lower in E2 than in E1. Whilst psychophysics generally involves the use of a higher amount of trials in order to accommodate for a small N [67], the results in E2 might also be caused by lack of power in the study. However, by looking at the effect sizes, there is a clear decrease in the effect of Task Type in E2 compared to E1.

The study of the effects of type of motion on peripheral acuity would also benefit from an investigation using low-level visual stimuli, such as Gabor patches; doing so would allow for the collection of baseline data that would be able to inform vision models such as those found in Mantiuk et al. [26] or Mantiuk et al. [19]. Decoupling the effects of the vestibular and visual system (e.g. through the use of motion platforms) could also give more insight into what causes AM to mask more degradation. This experiment could also be extended to consider multisensory inputs. Hulusic et al. [79] showed that pairing a walking animation with step sounds alters the perceived smoothness of the scene. Whilst our dependent variable only relies on the spatial domain, we believe this methodology can be extended to account for acoustic stimuli as well.

## Conclusion

This paper examined if artefacts caused by a dynamic FR algorithm driven by VRS4$\times$4 can be masked by task and movement type and are consistent across different visual behaviours (search and pursuit) typical of VR usage. We find that during visual search, active movement (walking) and more demanding tasks significantly increase participants’ tolerance to foveated rendering. The results of movement type seem to be consistent across visual behaviours (visual pursuit and search). Given the push towards VR and MR standalone devices and wireless tethering, we expect user activities to become more dynamic and involve more walking. Importantly, we show that directly available information about attentional load can also be used to fine-tune FR methods. We believe these findings could inspire more research that seeks to disentangle the complex interactions between these mechanisms and that these results can be derived and implemented in comprehensive vision models or directly into FR algorithms in the future.

## Data Availability

Data and code will be made available on request to the corresponding author.

## Footnotes

1 https://sites.manchester.ac.uk/VR2/.
